# From Bench to Bedside: How the Tumor Microenvironment Is Impacting the Future of Immunotherapy for Renal Cell Carcinoma

**DOI:** 10.3390/cells10113231

**Published:** 2021-11-19

**Authors:** Jonathan Anker, Justin Miller, Nicole Taylor, Natasha Kyprianou, Che-Kai Tsao

**Affiliations:** 1 Division of Internal Medicine, Icahn School of Medicine at Mount Sinai, New York, NY 10029, USA; Jonathan.Anker@mountsinai.org; 2 Division of Hematology and Medical Oncology, Tisch Cancer Institute, Icahn School of Medicine at Mount Sinai, New York, NY 10029, USA; Justin.Miller@mssm.edu (J.M.); Nicole.Taylor@mssm.edu (N.T.); 3 Tisch Cancer Institute, Icahn School of Medicine at Mount Sinai, New York, NY 10029, USA; Natasha.Kyprianou@mountsinai.org; 4 Department of Pathology and Molecular and Cell Based Medicine, Icahn School of Medicine at Mount Sinai, New York, NY 10029, USA; 5 Department of Urology, Icahn School of Medicine at Mount Sinai, New York, NY 10029, USA

**Keywords:** renal cell carcinoma, tumor microenvironment, immunology, immunotherapy

## Abstract

Immunotherapy has revolutionized the treatment landscape for many cancer types. The treatment for renal cell carcinoma (RCC) has especially evolved in recent years, from cytokine-based immunotherapies to immune checkpoint inhibitors. Although clinical benefit from immunotherapy is limited to a subset of patients, many combination-based approaches have led to improved outcomes. The success of such approaches is a direct result of the tumor immunology knowledge accrued regarding the RCC microenvironment, which, while highly immunogenic, demonstrates many unique characteristics. Ongoing translational work has elucidated some of the mechanisms of response, as well as primary and secondary resistance, to immunotherapy. Here, we provide a comprehensive review of the RCC immunophenotype with a specific focus on how preclinical and clinical data are shaping the future of immunotherapy.

## 1. Introduction

Renal cell carcinoma (RCC) is the eighth most prevalent cancer in the U.S with a lifetime risk of 1.7% and an estimated incidence of approximately 76,000 cases and 13,800 deaths in 2021 [1]. Interestingly, the disease displays a high degree of both inter- and intra-tumoral heterogeneity [2]. Histopathologically, RCC is divided primarily into the clear cell (ccRCC, 75%), papillary (pRCC, 15%), and chromophobe (chRCC, 5%) subsets. For ccRCC, although *VHL* alterations or chromosome 3p loss are the most common genetic alterations, there are many additional frequently observed alterations implicated in disease pathogenesis [3]. Genomic and transcriptomic analyses have highlighted this heterogeneity, defining distinct molecular RCC subtypes by various classification schemes [4,5,6], many of which carry unique characteristics, prognoses, and associations with response to specific treatments.

In parallel with a deeper understanding of RCC biology, management of the disease has significantly evolved in recent years, leading to improved patient outcomes [7]. This is, in large part, due to the resurgence of cancer immunotherapy. Previously, RCC was managed with cytokine-based therapies including IL-2 [8] and IFNα [9]; however, clinical benefit was largely limited to a very small subset of patients [8,10]. Subsequently, multiple tyrosine kinase inhibitors (TKIs) with anti-angiogenic properties provided an improved benefit to toxicity profile, leading to broader benefit for more patients. However, the duration of treatment response proved to be relatively modest [11]. Most recently, immune checkpoint inhibitors (ICIs) have emerged as an important modality for treating RCC (Table 1). These monoclonal antibodies are designed to antagonize inhibitory pathways that promote immunosuppression and tumor immune evasion. Specifically, ICIs targeting the PD-1/PD-L1 and CTLA-4/B7 axes have yielded great clinical success in many cancer types. PD-1 is a transmembrane protein expressed primarily on activated T cells, with additional expression on natural killer (NK) cells, B cells, dendritic cells (DCs), and macrophages. PD-1 binds to its ligand, PD-L1, which is often expressed on tumor cells and various antigen-presenting cells, leading to subsequent inhibition of T cell expansion and activity [12]. CTLA-4 is expressed on T cells after initial activation, as well as on regulatory T cells (Tregs), and serves to disrupt the crucial costimulatory signaling between CD28 and B7-1/CD80 and B7-1/CD86 needed for T cell activity [13,14]. While these interactions serve physiologic roles in dampening the adaptive immune response in chronic inflammatory conditions, in the setting of malignancy, they lead to an immunosuppressive microenvironment promoting tumor immune escape.

For patients presenting with localized RCC, surgery (either partial or radical nephrectomy) is standard of care and potentially curative. However, some will inevitably develop disease recurrence, which is associated with a poor prognosis [3]. In 2017, sunitinib was approved by the Federal Drug Administration in the adjuvant setting for patients at high risk for recurrence after nephrectomy, after demonstrating disease free survival (DFS) benefit in the S-TRAC trial [15]. However, utilization of such treatment approach has been largely underutilized, with lack of overall survival (OS) benefit in S-TRAC, lack of definitive benefit in other similar randomized studies [16], and the barrier of potential treatment toxicities. In the recently published phase III KEYNOTE-564 trial, adjuvant pembrolizumab (anti-PD-1) also demonstrated definitive DFS benefit in patients at high risk of recurrence after nephrectomy [17], offering another promising treatment option in this setting.

ICIs have also transformed the treatment landscape for patients with advanced and metastatic RCC. For patients previously treated with anti-angiogenic therapy, nivolumab (anti-PD-1) monotherapy proved superior to everolimus in patients with metastatic ccRCC who had progressed from prior anti-angiogenic therapy [18]. Pembrolizumab monotherapy has also shown promise as a first-line option, in both advanced ccRCC [19] as well as non-ccRCC [20]. While this single-armed approach provides clinical benefit for a subset of patients, many others do not benefit with meaningful or durable response.

Combination-based immunotherapy approaches have proven to be very effective for many cancer types, including RCC [21]. The synergy seen with combination ICIs may be due to the differential functions of the immunosuppressive pathways they antagonize. Dual ICI therapy results in a unique gene expression profile [22] and the expansion of specific T cell subsets [23] not seen with either PD-1/PD-L1- or CTLA-4-based monotherapies. In CheckMate 214, International Metastatic RCC Database Consortium (IMDC) intermediate/poor-risk ccRCC patients treated with nivolumab plus ipilimumab (anti-CTLA-4) had significant benefit in OS, PFS, quality of life, and subsequent treatment-free survival compared to those treated with sunitinib [24,25]. The median OS for those treated with the combination was 55.7 months, an unprecedented outcome that has not been previously seen with other therapies. However, 20% of patients experience primary progression, and, additionally, the durability of response varies widely. Thus, it is critical to better understand the unique properties of the tumor microenvironment (TME) to both identify mechanisms of resistance and develop optimal synergistic and novel combinatorial approaches.

Another effective combination approach is the use of an ICI concurrently with a TKI. Approved TKIs are generally multi-targeted, with predominant inhibitory activity on angiogenesis via the VEGF pathway. While these inhibitors have direct tumor cytotoxic activity by impacting the endothelium, they also display numerous immunomodulatory properties. VEGF functions in decreasing T cell infiltration and anti-tumor activity, while increasing intra-tumoral levels of Tregs, tumor-associated macrophages (TAMs), and myeloid-derived suppressor cells (MDSCs). Interestingly, transcriptomic analysis of sunitinib- or pazopanib-treated patients identified enrichment of PD-L1 and M2 TAMs in the patient cluster with the worst outcomes [26]. These preclinical findings make the ICI/TKI combination a promising therapeutic approach in patients with advanced ccRCC, leading to clinical benefit that has now been validated in several clinical trials [27,28,29,30,31].

**Table 1:** Approved immunotherapies for RCC.

Treatment(s)	Class	Setting	Indication	Key Data	Clinical Trial
Monotherapy					
Nivolumab	ICI	Second line	Advanced RCC after prior anti-angiogenic therapy	Nivolumab vs. Everolimus: • mOS: 25.0 mo (95% CI, 21.8–NE) vs. 19.6 mo (95% CI, 17.6–23.1) [HR 0.73; 98.5% CI, 0.57–0.93; *p* = 0.002] • mPFS: 4.6 mo (95% CI, 3.7–5.4) vs. 4.4 mo (95% CI, 3.7–5.5) [HR 0.88; 95% CI, 0.75–1.03; *p* = 0.11] • ORR: 25% vs. 5% [OR 5.98; 95% CI, 3.68–9.72; *p* < 0.001]	CheckMate 025 [18]
High-Dose IL-2	Cytokine	First line	Metastatic RCC	Proleukin: • ORR: 14% (90% CI, 10–19) • CR: 12 (5%) CR • PR: 24 (9%, median response duration 19.0 mo)	[8]
Combination Therapy					
Ipilimumab + Nivolumab	ICI + ICI	First line	Intermediate/poor-risk advanced RCC	Ipilimumab + Nivolumab vs. Sunitinib: • mOS: NR (95% CI, 28.2–NE) vs. 26 mo (95% CI, 22.1–NE) [HR 0.63; 99.8%, CI 0.44–0.89; *p* < 0.001] • mPFS: 11.6 mo (95% CI, 8.7–15.5) vs. 8.4 mo (95% CI, 7.0–10.8) [HR 0.82; 99% CI, 0.64–1.05; *p* = 0.03] • ORR: 42% (95% CI, 37–47) vs. 27% (95% CI, 22–31) [*p* < 0.001]	CheckMate 214 [24]
Nivolumab + Cabozantinib	ICI + TKI	First line	Advanced RCC	Nivolumab + Cabozantinib vs. Sunitinib: • Probability of OS at 12 mo: 85.7% (95% CI, 81.3–89.1) vs. 75.6% (95% CI, 70.5–80.0) [HR 0.60; 98.89% CI, 0.40–0.89; *p* = 0.001] • mPFS: 16.6 mo (95% CI, 12.5–24.9) vs. 8.3 mo (95% CI, 7.0–9.7) [HR 0.51; 95% CI, 0.41–0.64; *p* < 0.001] • ORR: 55.7% (95% CI, 50.1–61.2) vs. 27.1% (95% CI, 22.4–32.3) [*p* < 0.001]	CheckMate 9ER [31]
Pembrolizumab + Lenvatinib	ICI + TKI	First line	Advanced RCC	Pembrolizumab + Lenvatinib vs. Sunitinib: • mOS: NR (33.6–NE) vs. NR (NE–NE) [HR 0.66; 95% CI, 0.49–0.88; *p* = 0.0049] • mPFS: 23.9 mo (95% CI, 20.8–27.7) vs. 9.2 mo (95% CI, 6.0–11.0) [HR 0.39; 95% CI, 0.32–0.49; *p* < 0.0001] • ORR: 71% (95% CI, 66–76) vs. 36% (95% CI, 31–41) [*p* < 0.0001]	KEYNOTE-581/CLEAR [30]
Pembrolizumab + Axitinib	ICI + TKI	First line	Advanced RCC	Pembrolizumab + Axitinib vs. Sunitinib: • mOS: NR vs. NR [HR 0.53; 95% CI, 0.38–0.74; *p* < 0.0001] • mPFS: 15.1 mo (95% CI, 12.6–17.7) vs. 11.1 mo (95% CI, 8.7–12.5) [HR 0.69; 95% CI, 0.57–0.84; *p* < 0.001) • ORR: 59.3% (95%, CI 54.5–63.9) vs. 35.7% (95% CI, 31.1–40.4) [*p* < 0.001]	KEYNOTE-426 [27]
Avelumab + Axitinib	ICI + TKI	First line	Advanced RCC	Avelumab + Axitinib vs. Sunitinib: • 12 mo OS (PD-L1+): 86% vs. 83% (HR 0.78; 95% CI, 0.55–1.08; *p* = 0.14) • mPFS (PD-L1+): 13.8 mo vs. 7.2 mo [HR 0.61; 95% CI, 0.47–0.79; *p* < 0.001] • mPFS (overall population): 13.8 mo (95% CI, 11.1–NE) vs. 8.4 mo (95% CI, 6.9–11.1) [HR 0.69; 95% CI, 0.56–0.84; *p* < 0.001] • ORR (PD-L1+): 55.2% (95% CI, 49.0–61.2) vs. 25.5% (95% CI, 20.6–30.9)	JAVELIN Renal 101 [28]
IFNα-2a + Bevacizumab	Cytokine + VEGF inhibitor	First line	Metastatic RCC	IFNα-2a + Bevacizumab vs. IFNα-2a + Placebo: • mOS: 23.3 mo vs. 21.3 mo [HR 0.91; 95% CI, 0.76 to 1.10; *p* = 0.3360] • mPFS: 10.2 mo vs. 5.4 mo [HR 0.63; 95% CI, 0.52 to 0.75; *p* = 0.0001] • ORR: 31% vs. 12% [*p* < *0*.001]	AVOREN [9]

Immune checkpoint inhibitor (ICI), tyrosine kinase inhibitor (TKI), median overall survival (mOS), months (mo), confidence interval (CI), not estimable (NE), hazard ratio (HR), median progression free survival (mPFS), overall response rate (ORR), complete response (CR), partial response (PR), and not reached (NR).

## 2. Targeting the Tumor Microenvironment

In order to optimize the treatment approach for RCC, it is critical to understand the makeup of the TME that will determine the impact of each therapy. Transcriptomic analysis of the RCC immune infiltrate from The Cancer Genome Atlas (TCGA) database identified ccRCC as having the highest degree of total immune infiltration and T cell infiltration out of 19 cancer types [32], with an immunologically “cold” RCC TME rarely observed [33]. There are also various cell signaling pathways that serve to enhance or inhibit immune infiltration and function, including immune checkpoints such as PD-L1 and CTLA-4. Tumor mutational burden (TMB) has become another area of focus, as the generation of sporadic non-synonymous tumor-specific mutations, termed neo-antigens, can serve as the basis for enhanced tumor immunogenicity. RCC is unique in many of these regards, with a tumor immune composition and mutational patterns dissimilar to what are expected with an immunotherapy-responsive tumor type.

**Figure 1:**
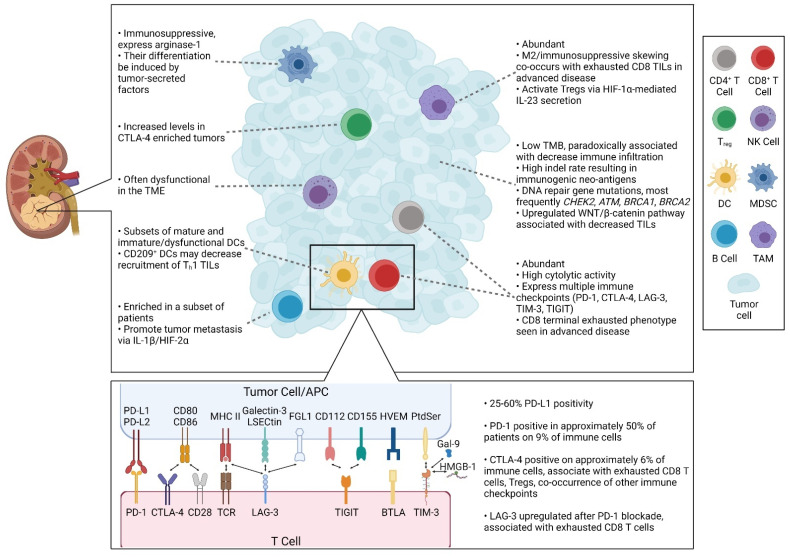
Representation of the RCC TME. Text highlighting key data regarding the infiltrating immune cells, tumor cells, and immune checkpoint molecules.

Importantly, in vivo, RCC typically exhibits a large degree of inter- and intra-tumoral heterogeneity [2]. Among the RCC subtypes, ccRCC demonstrates an increased immune response-related gene expression signature compared to pRCC and chRCC [34]. A separate taxonomic TCGA analysis that stratified RCC into nine distinct subtypes similarly found the ccRCC clusters to contain the greatest levels of total immune and T cell infiltration, as well as increased expression of the genes for CD3, PD-1, PD-L2, CTLA-4, CD134, and CD137 [5]. Mass cytometry-based clustering further highlighted the diversity of the immune makeup, identifying 22 distinct T cell and 17 distinct TAM phenotypes [35]. Here, we review the current understanding regarding the immunophenotype and relevant components that comprise the RCC TME (Figure 1).

### 2.1. Cellular Targets

#### 2.1.1. Tumor-Infiltrating Lymphocytes: CD8 T Cells, CD4 T Cells, Regulatory T Cells, and B Cells

Tumor-infiltrating lymphocytes (TILs) represent an integral and diverse component of the tumor immune infiltrate. Generally, CD8 T cells and Th1-differentiated CD4 T cells function to promote anti-tumor immunity, while Tregs and Th2 T cells are associated with immune evasion. Th17 CD4 T cells display mixed roles in the TME, and their differentiation exists within a homeostatic balance with Tregs. B cells also play conflicting roles, with the regulatory B cell subset specifically acting to modulate the anti-tumor immune response. RCC display a strong abundance of intra-tumoral T cells compared to other cancer types. Multiple TCGA analyses have identified ccRCC as having both the highest T cell infiltration score among 9 cancer types [32] and the highest proportion of “T cell-inflamed tumors” among 31 malignancies [36]. CD8 T cells, in particular, are enriched in the majority of cases [37,38]. Among the CD4 T cells, studies have reported an overall low level of Th2 T cells within RCC [32,37]. However, circulating CD4 T cells in the peripheral bloodstream appear to be skewed towards a Th2/Th17 phenotype, especially in higher stage tumors [39]. Among the RCC subsets, there may be a Th17 and Th2 predominance in chRCC and pRCC, respectively [34].

TILs are not only numerous within these tumors, they also appear to be highly functional. In one study quantitating immune cytolytic activity based on the expression level of the CD8 T cell cytolytic effector molecules granzyme A (*GZMA*) and perforin (*PRF1*), ccRCC ranked highest among 15 cancer types, and the scoring was markedly increased compared to pRCC and benign renal tissue [40]. Distinct CD8 and CD4 T cell populations display differential expression of the genes for activation and costimulatory markers including CD28, CD69, CD38, HLA-DR, OX-40, 4-1BB, ICOS, and CXCR3 and checkpoint markers such as PD-1, CTLA-4, and Tim-3 [35,37]. However, in more advanced and metastatic disease, RCC CD8 TILs shift towards a terminal exhausted phenotype, expressing multiple immune checkpoint molecules with restricted T cell receptor (TCR) diversity [41].

Tregs serve a physiologic role in promoting self-tolerance and maintaining immunological homeostasis under benign conditions. However, within the TME, they inhibit anti-tumor immunity through multiple mechanisms including via CTLA-4 and the production of immunosuppressive cytokines TGF-β and IL-10 [42]. The data regarding Tregs in RCC have been mixed. Compared to other cancer types, the RCC TME appears to contain a lower level of Tregs [32]. However, in the periphery, Treg levels have been observed in both greater [43] and lower [39] levels in RCC patients compared to healthy controls. Notably, patients with low levels of circulating Tregs appear to have these immunosuppressive cells comprise a greater proportion of TIL compartment within their tumors [43]. A similar pattern of decreased peripheral Tregs and increased tumor-infiltrating Tregs has been associated with higher tumor stage [39].

B cells play conflicting roles in modulating the tumor immune response. B cells and plasma cells are able participate in and promote antigen presentation, and produce antibodies and cytokines that enhance the anti-tumor immune response. However, subsets such as regulatory B cells can suppress the immune response through production of TGF-β, IL-10, and IL-35, as well as via antibodies that form tumorigenic immune-complexes [44]. While many RCCs do not contain a significant degree of B cells, a subset are highly infiltrated [45], with overall B cell levels documented at a frequency of approximately 4% in ccRCC [35]. Functionally, B cells recruited into renal tumors have been implicated in promoting tumor migration and metastatic potential via IL-1β/HIF-2α signaling [46]. B cell infiltration and B cell receptor (BCR) diversity may also hold poor prognostic implications for RCC [47].

#### 2.1.2. Dendritic Cells

Among the DC subsets, plasmacytoid DCs may be overrepresented in ccRCC [32,34]. Similar to Tregs, both myeloid DCs and plasmacytoid DCs have been observed in lower levels in the periphery but higher levels within the RCC TME, and many display an immature dysfunctional phenotype [48]. CD209+ DCs, in particular, may play a role in promoting tumor progression by decreasing the recruitment of Th1 T cells into the tumor [49]. Intra-tumoral DCs have similarly been linked as a prognostic marker for immunotherapy [50].

#### 2.1.3. Tumor-Associated Macrophages

Macrophages represent a diverse immune cell type, exemplified by the 17 distinct TAM phenotypes observed in RCC [35]. Immunophenotyping analysis identified M2-polarized TAMs, in addition to CD8 T cells, as the most abundant immune cell type within the RCC TME [51]. Discrepancies can exist between disease sites, as RCC metastatic lesions displayed a greater number of TAMs but a decreased immunosuppressive M2-skewing compared to the primary tumor [52], and intra-tumoral macrophages appear to be decreased after treatment with bevacizumab [53]. TAMs are intricately involved in crosstalk with many other cell types, and, in RCC, evidence suggests that they activate Tregs via HIF-1α-mediated IL-23 secretion triggered by tumor glutamine consumption [54]. A more M2-skewed TAM phenotype appears to be enriched in advanced and metastatic RCC, co-occurring with terminally exhausted CD8 TILs in a hypothesized “immune dysfunction circuit” [41].

#### 2.1.4. NK Cells

NK cells are another abundant and cytotoxic immune cell type in RCC [55], documented at a frequency of approximately 9%. Among the RCC subtypes, pRCC [34] may contain the strongest proportion of NK cells in both the primary tumor and metastatic lesions [56]. Renal tumor cells may induce NK cell dysfunction through multiple mechanisms involving the diacylglycerol kinase [57], mitogen-activated protein kinase (MAPK/WEK) [57], and TGF-β/SMAD [58] signaling pathways.

#### 2.1.5. Myeloid-Derived Suppressor Cells

MDSCs, characterized as granulocytic/polymorphonuclear (G/PMN-MDSCs) or monocytic (M-MDSCs) based on their cell lineage of origin, further suppress the anti-tumor immune response through multiple mechanisms including the production of arginase-1, reactive oxygen species, TGF-β, and IL-10 [59]. In RCC, total MDSCs, G-MDSCs, and immature-MDSCs have been correlated with increasing tumor grade and stage [60], and are functionally immunosuppressive through arginase-1 production [61]. MDSC generation may be tumor-induced, as exposure to conditioned media from RCC cell lines resulted in functionally immunosuppressive M-MDSC differentiation [62]. Interestingly, preclinical RCC data have demonstrated that inhibitors to CXCR2 [63], IL-1β [63], class I histone deacetylase (HDAC) [64], and HMGB1 [65] can slow tumor growth and potentially synergize with anti-PD-1 blockade in an MDSC inhibition-dependent manner. Finally, anti-angiogenesis TKIs such as sunitinib can also suppress MDSC activity [66], but this immunomodulatory ability may be mitigated by intra-tumoral GM-CSF [67].

### 2.2. Extracellular Targets

#### 2.2.1. Tumor Mutational Burden

High TMB is a common feature shared among the majority of tumor types known to respond to ICIs [68,69,70,71]. RCC is unique in that despite its high degree of immune infiltration, these tumors have proven to contain a relatively low mutational burden. RCC has a mutation rate of approximately 1.1 mutations/megabase (mut/Mb), even lower (<1 mut/Mb) in chRCC [72,73]. That places RCC lower than many of its ICI-approved counterparts, including melanoma, non-small-cell lung cancer, urothelial carcinoma, colorectal cancer, cervical cancer, head and neck cancer, endometrial cancer, and hepatocellular carcinoma. ccRCC demonstrates a paradoxical correlation in which higher TMB is associated with decreased survival and decreased levels of intra-tumoral CD8 T cells, M1 and M2 macrophages, CD4 memory resting T cells, and DCs [74]. Separate database analysis correlated high TMB in ccRCC with lower gene expression for TILs, immune checkpoints, cytokines, and other pro-inflammatory genes, and high TMB in pRCC was associated with low PD-L1 levels, Tregs, and expression of pro-inflammatory genes [75].

#### 2.2.2. Neo-Antigens

Somatic tumor alterations occasionally result in the production of neo-antigen peptides that contain unique tumor-specific and immunogenic epitopes. Neo-antigens strongly correlate with TMB, and represent a mechanistic bridge linking TMB with tumor immunogenicity. One reason RCC may not abide by this trend involves the patterns in which RCC undergoes genetic alterations. RCC (including ccRCC, pRCC, and chRCC) demonstrated the highest proportion and number of insertion-deletion (indel) mutations among a pan-cancer study, 2.4-fold greater than the average cancer rate. Indels, especially those that create open reading frames, are associated with a higher rate of neo-antigen formation than single nucleotide variants (SNVs), and may be highly immunogenic, with RCC-specific neo-antigens associated with higher expression of genes for antigen presentation and CD8 [76].

Another explanation for this discordance may be due to tumor immunity within the RCC TME. In an analysis of predicted neo-antigen burden based on non-silent point mutations, ccRCC demonstrated one of the lowest ratios of observed:predicted neo-epitopes. Authors hypothesized ccRCC to represent an “immune-susceptible” tumor type. Given its known high cytolytic activity, the lower than expected neo-antigen load may represent spontaneous tumor surveillance resulting in clonal elimination of neo-epitope expressing cancer cells [40].

#### 2.2.3. DNA Repair

Aberrations in DNA repair pathways is a hallmark of malignancy [77]. DNA repair deficiencies lead to a hypermutable state resulting in increased formation of neo-antigens, and may serve as a biomarker of ICI efficacy [78,79]. In RCC, DNA repair gene alterations have been documented at frequencies of approximately 19–25% [80,81], with *CHEK2*, *ATM,* and *BRCA1,* and *BRCA2* most commonly implicated [80,82]. Up to 26 DNA repair genes have displayed differential expression in ccRCC compared to benign tissue, six of which were identified as potential prognostic biomarkers (*ISG15*, *RAD51AP1*, *SFRP2*, *SLFN11* as high-risk genes; *SPATA18*, *VAV* as low risk genes). After risk stratifying patients based on DNA repair gene expression, high-risk tumors contained increased expression of immune checkpoints (genes for PD-1, LAG-3, CTLA-4, and TIGIT) and were enriched for neutrophil activity-related gene expression pathways [83].

#### 2.2.4. Tumor and Immune Metabolism

Cancer and immune cell metabolism is another significant component of the TME capable of modulating anti-tumor immunity and impacting response to immunotherapy [84]. RCC is a particularly metabolically-driven disease process, with genetic alterations resulting in metabolic changes allowing tumor cells to survive in hypoxic conditions [85]. Metabolomic analyses have uncovered specific metabolic reprogramming that occurs within RCC tumor cells relating to glucose uptake and glycolysis, amino acid utilization, and fatty acid metabolism [85,86,87,88,89], with prognostic and therapeutic implications [34,87,90,91,92]. Specifically, the kynurenine/tryptophan ratio negatively correlated with response to nivolumab, representing a potential mechanism of ICI resistance with implications for combination therapy with indoleamine 2,3-dioxygenase 1 (IDO1) inhibition [93]. Single-cell transcriptomic analysis identified a distinct RCC tumor cell subset with metabolic plasticity as determined by increased glycolysis, oxidative phosphorylation, and fatty acid metabolism, which was associated with 9p21.3 deletion and differential expression of immune checkpoint molecules [94]. Regarding immune cell metabolism, TILs from RCC patients may display metabolic dysfunction with aberrant glucose utilization, increased reactive oxygen species, and a distinct mitochondrial morphology [95].

### 2.3. Immune Checkpoints

ICIs targeting the PD-1/PD-L1 interaction have become a mainstay of treatment for many cancers. Reported PD-L1 positivity in RCC ranges from 25% up to 60% [96], with approximately 32% of patients having ≥5% positivity [97]. PD-1 positivity is seen on approximately 9% of RCC-infiltrating mononuclear cells [98] and in 57% of patients, commonly co-occurring in tumors expressing PD-L1 [99]. When comparing the primary renal tumor to metastatic lesions, site-based discrepancies for PD-L1 expression was observed in approximately 20% of cases, primarily involving PD-L1 positive primary tumors losing expression at distant sites [100]. Overall, PD-L1 positivity has been associated with more aggressive disease, increased tumor size and grade, and poor prognosis [101,102,103].

A number of additional immune checkpoints have been prime targets for therapeutic investigation, including CTLA-4, PD-L2, LAG-3, TIM-3, and TIGIT, which are upregulated in ccRCC [104]. CTLA-4, most notably, can be found on approximately 6% of RCC-infiltrating mononuclear cells [98]. Tumors with increased CTLA-4 levels have been linked to a more immune enriched TME with differential cytokine and chemokine expression [104], but those immune cells appear to comprise an exhausted CD8 T cell and Treg predominant phenotype, with increased expression of the checkpoints PD-L1, PD-1, LAG-3, IDO1, and TIGIT [105]. Interestingly, CTLA-4 expression may be linked to TMB via *BAP1* mutations [105], and the miRNA miR-20b-5p was identified as a potential target of CTLA-4 with positive prognostic associations in RCC [104]. Importantly, CTLA-4 blockade via ipilimumab has been approved for the treatment of ccRCC in combination with nivolumab [24].

LAG-3 is upregulated in RCC compared to benign tissue [106]. Its expression appears to be under epigenetic regulation by DNA methylation, and levels correlated with TIL- and IFNγ-related gene expression [107]. TIM-3 is also associated with immune infiltration and cytokine expression in ccRCC [104]. LAG-3 and TIM-3 are seen on both CD4 and CD8 TILs in RCC. LAG-3, in particular, is often co-expressed on TILs with PD-1, with PD-1 blockade upregulating LAG-3 and in vitro data suggesting promise for this therapeutic combination [108]. TIGIT is detectable in approximately 75% of ccRCC tumors, primarily on tumor-infiltrating T cells and NK cells [109]. TIGIT expression is greater in tumor regions than adjacent normal tissue, with levels higher than those quantified for PD-1, and its expression correlated with that of CD3ε [110] but not with tumor grade or stage [109]. Further, an inhibitory TIGIT antibody demonstrated preclinical efficacy in a murine kidney tumor model in a T cell-dependent manner [111]. A histological analysis of LAG-3, TIM-3, and TIGIT identified distinct intra-tumoral phenotypes dominated primarily by a single checkpoint. In this study, the LAG-3 cluster was associated with a CD39+ exhausted CD8 T cell- and macrophage-dominant phenotype, and the TIGIT cluster with higher CTLA-4 expression [112]. Finally, multiple immune checkpoints, including PD-L1, PD-1, CTLA-4, LAG-3, TIM-3, and TIGIT, have been associated with genomic instability [113], long non-coding RNAs [114], *TNFRSF9* expression and TNFRSF9+ CD8 TILs [115], and CXCL13+ CD8 TILs [116] in RCC.

## 3. Current State of Biomarkers for Immunotherapy

**Figure 2:**
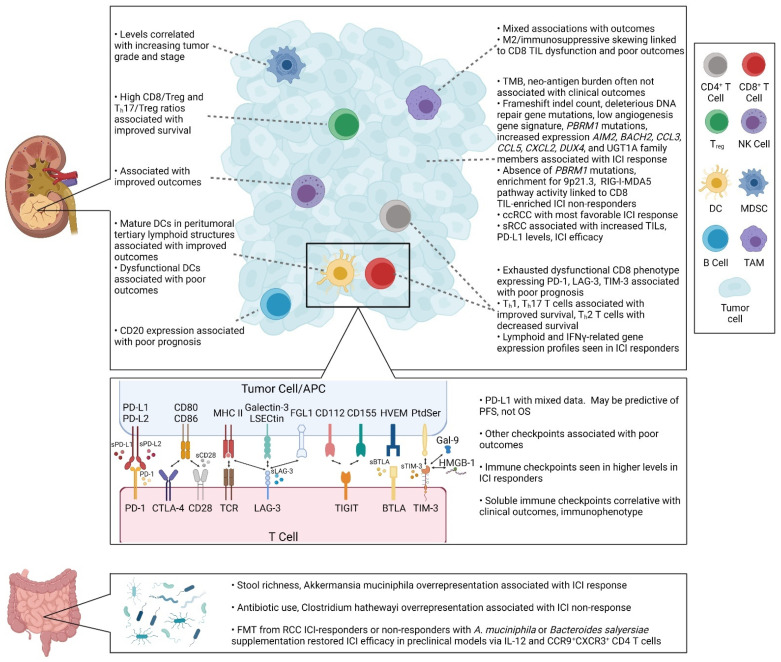
Biomarkers for immunotherapy in RCC. Text highlighting key biomarker-related data regarding immune cells, tumor cells, immune checkpoint molecules, and the microbiome.

Clinically validated biomarkers are critical for optimizing immunotherapy use with accurate patient selection. Generally, biomarkers are either prognostic or predictive, with the former used to identify the risk of an outcome irrespective of treatment, and the latter of which may be utilized to identify responding patients to a specific treatment. One of the challenges in identifying biomarkers, especially predictive biomarkers, is the great deal of both inter- and intra-tumoral heterogeneity in RCC, as discussed above. Single biomarkers may not be generalizable to the entire RCC population or even to the entirety of a single patient’s disease. More accurate and clinically meaningful biomarkers may require novel molecular- or immunological-based stratifications that account for the complexity of the TME. Here, we provide an overview of the existing data regarding RCC immune-related biomarkers (Figure 2). It is important to keep in mind that these biomarkers predominantly fall within the prognostic grouping, and have not been validated for patient selection in the clinical setting.

### 3.1. Single Immune Checkpoints

PD-L1 expression is utilized for patient selection for ICIs in multiple cancers. However, its role as a biomarker remains controversial, with confounding variables including differential sensitivities between the multiple immunohistochemistry assays, differences in PD-L1 positivity cutoff values, cell types included in the scoring (tumor ± immune cells), intra-tumoral heterogeneity, and biopsy timing and specimen quality [117]. Given the lack of definitive data to support its use in RCC, routine use of PD-L1 expression to select patients for ICI treatment is currently not standard of care. In CheckMate 214, nivolumab and ipilimumab provided OS and ORR benefit among intermediate- and poor-risk patients regardless of PD-L1 expression. However, increased PFS was seen among patients with PD-L1 ≥1% [24]. Further, for atezolizumab and bevacizumab, increased PFS trended with PD-L1 expression level in IMmotion150 [118], and clinical benefit increased in a PD-L1 level-dependent manner ranging from <1% to ≥10% in IMmotion151 [29]. On the contrary, ICI efficacy was independent of PD-L1 status in KEYNOTE-426 [27], JAVELIN Renal 101 [28,119], and CheckMate 025 [18]. A meta-analysis comparing these six trials concluded that PD-L1 expression may be predictive of PFS but not OS [96].

Although not clinically validated, several other immune checkpoints have also been investigated as potential biomarkers to select for ICI use for RCC patients. Increased expression of the genes for PD-L2, PD-1, CTLA-4, and HHLA2 have been associated with worse OS, PFS and DFS [98,104,105,120,121], with concurrently high CTLA-4 and PD-1 expression conferring an especially high-risk state [98]. Conversely, low expression of PD-L1 and TIM-3 have been linked to worse prognosis [104]. Specifically on tumor-infiltrating mononuclear cells, PD-1 and CTLA-4 expression were associated decreased OS [98], with PD-1 linked to higher tumor grade, tumor stage, and cancer-specific death [99]. In terms of response to ICI, higher levels of PD-1, PD-L1, LAG-3, IDO1, ICOS, and BTLA were associated with patients responding to nivolumab treatment [122]. When analyzing the soluble form of immune checkpoints in the periphery of ccRCC patients, correlations were observed between soluble BTLA and TIM-3 and decreased survival, PD-L2 with recurrence, TIM-3 and LAG-3 with advanced stage, and LAG-3 and CD28 negatively correlated with T cell cytolytic activity [123]. Finally, tumor stratification based on DNA repair gene expression identified high-risk tumors with increased expression of the checkpoints PD-1, LAG-3, CTLA-4, TIGIT [83], and classifying tumors by high expression of the immune checkpoints PD-1, PD-L1, PD-L2, and LAG-3, as well as the absence of mature DCs, was associated with poor prognosis even in the setting of CD8 T cell infiltration [121].

### 3.2. Immune Cells and Immune Gene Signatures

An overarching pattern across cancer types is the association between increased TILs and improved outcomes and response to immunotherapy [124]. However, the data for TILs in RCC have been mixed, with multiple studies linking infiltration with poor [37,56,74,125,126,127,128] and improved [32] outcomes. Deeper analyses have identified that poor prognosis was specifically associated with exhausted polyclonal CD8 T cells expressing immune checkpoints such as PD-1, TIM-3, and LAG-3 and displaying decreased cytotoxic functionality [121,127]. In addition, these associations must be considered in the context of the disease state being studies, as increasing TILs are seen in higher grade tumors [37,128], with terminally exhausted CD8 TILs with restricted TCR diversity populating more advanced and metastatic RCC [41]. Among the T cell subsets, Th1 T cells, Th17 T cells, and high Th17/Treg and CD8/Treg ratios have been associated with improved survival [32,34,37], while Tregs (circulating, tumor infiltrating, and specifically ICOS+ Tregs) and Th2 T cells were associated with decreased survival [32,34,43,121,127].

In CheckMate 009, pre-treatment analysis of nivolumab responders demonstrated 311 differentially expressed genes, including higher immune response in silico (IRIS) transcripts of myeloid and lymphoid lineages, IFNγ and IFNα response gene expression, and a higher T cell CD3TCR expression score [129]. IMmotion150 and IMmotion151 biomarker analyses identified myeloid- and angiogenesis-related gene expression patterns, as well as the expression of T effector/IFNγ response genes to be associated with PD-L1 expression and PFS after ICI [118,130]. In JAVELIN Renal 101, CD8 T cells correlated with PFS by gene expression deconvolution, but not by IHC [119]. Interestingly, among circulating immune cells, a low baseline level of PD-1 + CD69+ exhausted activated CD4 T cells and a high baseline level of CD244^+^ exhausted CD4 T cells were associated with higher risk of progression after nivolumab [131].

Among the other immune cell types, NK cells, CD11c+ TAMs, and mature DCs have been associated with improved outcomes [37,56,74,121,132], and CD206+ TAMs, CD20+ B cells, and DC-LAMP+ and dysfunctional DCs with decreased prognosis [45,56,121,127,132]. Immunosuppressive M2-phenotype macrophages in advanced disease may exist in a immune dysfunction circuit with terminally exhausted CD8 TILs, which together was linked to poor outcomes [41]. In an analysis of 14 cancer types, poor survival with high B cell infiltration was specifically associated with high expression of *MS4A1* (CD20) for ccRCC [45]. With respect to DCs, mature functional DCs that aggregate in peritumoral tertiary lymphoid structures have been associated with better prognosis [121], in contrast to poorer outcomes seen tumors with dysfunctional DCs [127]. Further, utilizing patient-derived xenograft models, an inflamed TME RCC subtype, with enrichment for the stromal compartment and multiple immune-infiltrating cell types (CD8 T cells, Th1 T cells, Tregs, NK cells, neutrophils, and TAMs), was associated with decreased survival [133].

### 3.3. TMB, Neo-Antigens, and DNA Repair

As described above, biomarkers such as TMB and neo-antigen burden do not hold the same prognostic associations in RCC as they do in other cancer types. In general, these correlations with clinical outcomes are strongest in tumor types in which CD8 TILs directly correlate with neo-antigen load [134]. In the IMmotion150 and JAVELIN Renal 101 trials, there was no association with TMB or neo-antigen burden and PFS [118,119]. This has been corroborated in additional studies [33], but, interestingly, an abstract reported that frameshift indel count did correlate with OS after PD-1 blockade [51]. DNA damage repair deficiency has also been associated with improved OS after immunotherapy for RCC [80].

### 3.4. Genomic Profiles

Genomic analyses of the ICI trials have also elucidated gene expression patterns that may have biomarker implications for RCC going forward. Response to anti-PD-1 treatment has been associated with loss of function mutations in *PBRM1*, a member of the SWI/SNF chromatin remodeling complex and one of the most commonly altered ccRCC genes seen in up to 50% of cases [135], resulting in aberrant expression of immune-related, JAK/STAT, and hypoxia pathways [136,137]. An immune-related prognostic model with *PBRM1* mutations identified a high-risk subgroup with increased Tregs, TAMs, and immune checkpoints including PD-1, CTLA-4, LAG-3, TIGIT, and CD47 [138]. Interestingly, absence of *PBRM1* mutations and enrichment for loss of 9p21.3, which contains antigen presenting machinery and HLA class II genes, were observed in many tumors with high levels of CD8 TILs, and may explain some of paradoxical correlations and ICI resistance seen in these patients [33]. Mechanistically, the lesser immunogenicity associated with loss of *PBRM1* may be attributed to decreased IFNγ-STAT1 signaling impacting the expression of IFNγ target genes [139]. As a biomarker, *PBRM1* mutations may prove useful for predicting response to nivolumab, especially in the second- or later-line setting for ccRCC patients [140].

Response to nivolumab has also been linked to increased expression of *AIM2* (inflammasome) [129], *BACH2* (CD4 differentiation regulator), *CCL3*, and UGT1A family members (metabolic and solute transport pathway) [141]. The JAVELIN Renal 101 biomarker analysis identified that PFS was associated with a 26-gene panel, termed “Renal 101 Immuno signature”, including genes involved in T cell activation, NK cell-mediated toxicity, the chemokines CCL5 and CXCL2, and *DUX4* which is involved in HLA suppression and antigen presentation [119]. In addition a low angiogenesis gene signature correlated with increased PFS after atezolizumab plus bevacizumab [130]. The immunosuppressive WNT/β-catenin signaling pathway also appears active in RCC, and was associated with decreased TILs [36,129] and higher TMB tumors with poor outcomes [74]. Interestingly, some of the anti-tumorigenic immunomodulatory properties of the TKI pazopanib may be due to its inhibitory activity on the β-catenin pathway [142].

Molecular subtypings have also been identified that associate with immunotherapy response. Taxonomic analysis from TCGA stratified RCC into nine distinct subtypes based on DNA mutations, copy number variations (CNVs), DNA methylation, and gene expression. Among the three ccRCC subtypes, high expression of the genes for PD-1, CTLA-4, and TLR9 were associated with decreased survival, while PD-L1 was linked with better outcomes (though per authors this may be confounded by the loss of 9p, which contains the PD-L1 locus, leading to more aggressive disease) [5]. The BIONIKK trial is implementing a 35-gene expression panel that will stratify patients in one of four molecular subtypes for treatment assignment with either ICI-, ICI/ICI-, or ICI/TKI-based regimens [143]. Prospective trials such as this may prove fruitful for identifying predictive biomarkers to influence patient selection.

### 3.5. Microbiome

The microbiome plays an integral role in tumorigenesis, systemic immunity, and the host anti-tumor immune response in the setting of immunotherapies. In RCC, antibiotic use surrounding ICI administration has been associated with decreased PFS, leading to overrepresentation of species including *Clostridium hathewayi* [144,145]. TKI use can also alter the microbiome composition in RCC patients [144]. Metagenomic sequencing of RCC fecal samples linked stool richness by gene count of metagenomic species levels with ICI response, specifically in patients with overrepresentation of the commensal *Akkermansia muciniphila* [144,145,146]. Interestingly, fecal microbiota transplantation (FMT) from RCC ICI responders, or FMT from non-responders with additional *A. muciniphila* or *Bacteroides salyersiae* supplementation, restored ICI activity in antibiotic-treated and germ-free mice in an IL-12- and CCR9 + CXCR3+ CD4 T cell-dependent manner [144,145]. Further, RCC patients given probiotic supplementation including *Bifidobacterium animalis*, which is depleted in RCC patients, led to increased levels of the bacteria within the gut microbiome. While there was no effect on outcomes for the probiotic with TKI treatment, responding patients had higher levels of *A. muciniphila* and *Barnesiella intestinihominis* [147].

### 3.6. Clinical Phenotypes

Clinical features associated with response to ICI in RCC include a high neutrophil-to-lymphocyte ratio (NLR), both at baseline and after treatment [148], IMDC poor/intermediate-risk patients, and the Memorial Sloan Kettering Cancer Center (MSKCC) poor-risk group [149,150], although between the MSKCC risk groups there was no difference in TMB or the expression cytolytic or immune checkpoint genes [151]. On the contrary, high systemic immune-inflammation index (SII), low BMI < 25 kg/m^2^, and older age ≥ 70 years old have been linked with decreased OS with nivolumab [152].

As expected based on the immune profile of non-ccRCCs, these tumors demonstrate lesser responses to ICI treatment than ccRCC [153]. Among the RCC subtypes, sarcomatoid dedifferentiation (sRCC) is seen in approximately 4% patients, and is associated with more aggressive disease [154]. sRCC tends to have higher PD-1/PD-L1 levels and CD8 TIL density in comparison to non-sarcomatoid RCC [99,155], the former of which may be attributed to 9p24.1 amplification seen in approximately 6% of sRCC [156]. Similarly, trials have consistently demonstrated increased PD-L1 positivity and expression of IFNγ- and T cell effector-related genes for sRCC [130,157,158,159]. Additionally, first-line ICI in patients with metastatic ccRCC with sarcomatoid differentiation has been associated with an excellent clinical response [160].

## 4. Future Directions: How Can We Develop Better Treatments by Creating a More Favorable Anti-Tumor Immune Microenvironment?

The future of immunotherapy for RCC (Table 2) must build upon the foundational knowledge of the TME and the mechanisms of both response and primary and secondary resistance. While immunotherapy is now an established treatment option for many RCC patients, only a minority of patients respond to monotherapy ICI. The future lies in a combination-based multi-modal approach to best optimize the immunomodulation required for clinical benefit. As such, the combinations of PD-1 with CTLA-4 blockade and ICIs with TKIs have already garnered approval for RCC, and many more approaches are currently under active investigation.

### 4.1. ICIs

Regarding the ICIs, there are numerous clinical questions yet to be answered. That includes the context in which to best administer ICIs, as it remains to be seen whether immunotherapy has a role in the neoadjuvant and adjuvant setting. The KEYNOTE-564 trial recently reported increased DFS with adjuvant pembrolizumab in patients at high risk of recurrence [17]. Ongoing investigations are assessing the efficacy of adjuvant nivolumab ± ipilimumab (CheckMate 914) [161], adjuvant durvalumab (PD-L1) ± tremelimumab (CTLA-4) (RAMPART) [162], as well as durvalumab and tremelimumab in the neoadjuvant and adjuvant setting [163].

Another ongoing question regards the use of ICI or combination therapy as first-line versus second-line treatment. In KEYNOTE-427, first-line pembrolizumab demonstrated promising activity in both advanced ccRCC [19] and non-ccRCC [20]. While nivolumab is approved in the post-anti-angiogenic therapy setting, its role as a first-line treatment is currently under investigation in multiple trials [164,165].

The optimal duration of therapy, potential maintenance dosing, and subsequent treatment regimens is also unknown. In the OMNIVORE trial, nivolumab responders will undergo observation without active treatment, while non-responders will be switched to ipilimumab. Preliminary reports suggests a low rate of response conversion after subsequent ipilimumab therapy [166]. In HCRN GU16-260, salvage nivolumab plus ipilimumab dual ICI after non-response to first-line nivolumab demonstrated an 11% PR rate [164]. The sequential regimen of nivolumab followed by combination nivolumab plus ipilimumab is being evaluated in UNISON/ANZUP 1602 [167]. TITAN-RCC is testing the potential efficacy of a nivolumab and ipilimumab “boost” after nivolumab monotherapy [165].

PD-1/PD-L1 blockade is also in combination trials with other checkpoint inhibitors, including those targeting CTLA-4 [168], LAG-3 [169], and TIM-3 [170], as well as the combination of concurrent PD-1 (MEDI0680) and PD-L1 (durvalumab) antagonism [171]. In another approach, RCC is among multiple cancer types in trial for CA-170, an oral small molecular inhibitor of PD-L1 and VISTA [172]. Finally, a more biomarker driven approach may ultimately prove necessary to best select patient subpopulations most appropriate for specific treatment, and the ongoing BIONIKK trial is utilizing a 35-gene expression panel to stratify patients into one of four molecular subtypes, based on which patients will be treated with either nivolumab monotherapy, combination nivolumab and ipilimumab, or combination ICI followed by sunitinib or pazopanib [143].

### 4.2. TKIs and HIF-2α Inhibitors

Anti-angiogenesis TKIs have a clear role in the treatment of RCC and display multi-faceted immunomodulatory properties. Current clinical combination approaches in development include: TKI with a single ICI in multiple disease settings [31,173,174,175,176,177], triple therapy with TKI plus dual ICI/ICI [176,178], adding cabozantinib to nivolumab as maintenance in patients who had clinical benefit to first-line ipilimumab plus nivolumab [179], and using ICI/TKI as a second-line treatment for patients who previously received ICI [180]. In addition, TKI combination approaches are also in development for those with non-ccRCC, including combining the MET-TKI savolitinib with durvalumab [181]. The combination of lenvatinib and pembrolizumab has definitively demonstrated superiority to sunitinib in the first-line setting for metastatic ccRCC [30], and the same combination is also under investigation as a salvage option after progression with anti PD-1/PD-L1 treatment [182].

HIF-2α inhibitors have recently gained traction as a promising avenue for RCC treatment. *VHL* inactivation leads to constitutive HIF-2α activity, thereby promoting tumorigenesis and immune evasion with upregulation of various downstream targets, including VEGF. HIF-2α inhibitors, such as PT2385 and belzutifan (MK6482/PT2977) have now reached phase III clinical trials for RCC [183], and are being tested in combination with ICIs [184]. HIF-2α inhibition may modulate the immune microenvironment, decreasing immunosuppressive myeloid cells while increasing infiltration by mature DCs [185].

### 4.3. Cellular Therapies

Adoptive cell therapies, which have yielded clinical success in multiple cancer types, are also in trial for RCC. Chimeric antigen receptor (CAR) T cell therapy involves autologous administration of patient-derived T cells engineered ex vivo with synthetic receptors allowing them to specifically target and eliminate tumor cells. CAR T cells under investigation for RCC include those targeting VEGFR2, c-Met, Ror2, and CD70 (trial currently suspended), as well as the previous target carboxy-anhydrase-IX (terminated due to hepatotoxicity [186]). Another cellular approach involves dendritic cell stimulation of cytokine-induced killer (D-CIK) cells with ex vivo expansion of T cells exposed to pembrolizumab, which is in trial in combination with axitinib.

### 4.4. Vaccines

Vaccines have thus far demonstrated limited efficacy for RCC. In the IMPRINT trial, the multi-peptide vaccine IMA901 did not confer added benefit to sunitinib as a first-line therapy [187]. Rocapuldencel-T (AGS-003), an autologous dendritic cell-based vaccine, demonstrated benefit in early phase trials [188], but the phase III ADAPT trial in combination with sunitinib was terminated early after interim analysis suggested no benefit for OS [189]. Ilixadencel (INTUVAX), in which activated allogeneic DCs are injected intra-tumorally, induced an anti-tumor immune response in early phase trial [190], with promising results from the interim analysis of the phase II MERECA trial involving pre-nephrectomy vaccine administration with adjuvant sunitinib [191]. Personalized neo-antigen vaccines based on patient-specific mutations have also been of interest, and related trials with GEN-009 [192], NeoVax (with and without ipilimumab), VB10.NEO (with the IL-2 agonist bempegaldesleukin) [193], and cevumeran (with atezolizumab) are currently underway for RCC. Additional vaccine approaches include a DC tumor fusion with GM-CSF, a recombinant vaccinia virus (with cemiplimab, PD-1) [194], a whole cell autologous and allogenic approach, the TLR3 agonist polyICLC (with tremelimumab and durvalumab) [195], and a prostate-specific membrane antigen (PSMA)-based vaccine.

### 4.5. Cytokine Stimulation: IL-2

IL-2 has long been tested as an immunotherapy for RCC, given its ability to promote expansion of T cells and NK cells. The combination of high dose IL-2 and pembrolizumab has shown preliminary activity [196], and is also under investigation with nivolumab [197]. Later-generation IL-2 therapies, such as the bempegaldesleukin (BEMPEG/NKTR-214), preferentially act through the CD122 pathway, thereby limiting the Treg-promoting function of IL-2. In the PIVOT-02 trial, the combination of bempegaldesleukin plus nivolumab conferred ORRs of 71.4% as a first-line and 28.6% as a second-line treatment for immunotherapy-naïve RCC, leading to increased levels of CD8 TILs [198]. The phase III PIVOT-09 trial comparing this combination with TKIs in the first-line setting is currently active [199].

### 4.6. Microbiome

The microbiome is another potential target for immunomodulation. In RCC, antibiotic use has been associated with poorer outcomes with ICIs [144,145], with *A. muciniphila* and *B. salyersiae* identified as potential biomarkers or therapeutic targets [144,145,146]. Regarding clinical trials, probiotic supplementation of *B. animalis* in RCC patients altered the makeup of the microbiome but did not impact TKI efficacy [147]. Ongoing trials are further investigating the evolution of the microbiome of RCC patients over the course of various treatments, the efficacy of FMT from RCC ICI responders, and the ability to use FMT to mitigate the toxicity from combination ICI treatment.

### 4.7. Other Immunomodulators

Investigators have also attempted to target the immunosuppressive TGF-β pathway, using the ALK1 inhibitor dalantercept combined with axitinib [200,201], but the drug was subsequently discontinued after the DART study failed to demonstrate clinical benefit. TRC105, which targets endoglin, an accessory receptor for TGF-β, is currently being tested with axitinib [202]. Other immunomodulatory targets in clinical trial with ICIs include inhibitors to IDO1 (including the phase III KEYNOTE-679/ECHO-302 trial), CCR4, CCR2/5, CD73, HDAC [203,204], histone methyltransferase EZH1/2, PARP [205], mTOR, glutaminase, the adenosine A2A receptor [206] (an Adenosine Signature biomarker was identified in responders [207]), and the p53-MDM2 interaction. Immunomodulatory agonists in combination with ICIs are targeting 4-1BB [208], OX-40, CD27, ICOS [209], and GITR. Lastly, ICIs are being evaluated with platinum-based chemotherapy [205] and guadecitabine (with reported decreased MDSCs and Tregs in responders, and Th17 T cells associated with immune-mediated toxicity [210]), stereotactic body radiotherapy [211], and cryoablation.

**Table 2:** Ongoing and completed clinical trials for non-approved immunotherapies for RCC.

Treatment(s)	Phase	Setting, Patient Population	Key Data *	Clinical Trial
ICIs				
Nivo ± Ipi	III	First-line, intermediate/poor-risk advanced ccRCC		NCT03873402
Pembro	III	Adjuvant, ccRCC with high-risk, intermediate–high-risk, or M1 NED	Pembro vs. Placebo: • 24 mo DFS: 77.3% vs. 68.1% [HR 0.68; 95% CI, 0.53–0.87; *p* = 0.002]	KEYNOTE-564, NCT03142334 [17]
Nivo ± Ipi	III	Adjuvant, high-risk ccRCC		CheckMate 914, NCT03138512 [161]
Durva ± Treme	III	Adjuvant, RCC with high/intermediate risk of relapse		RAMPART, NCT03288532 [162]
Pembro	II	First-line, advanced ccRCC or non-ccRCC	• mOS: NR • mPFS: 7.1 mo (95% CI, 5.6–11.0) • ORR: 36.4%	KEYNOTE-427, NCT02853344 [19,20]
Nivo followed by Nivo + Ipi	II	First-line and salvage, advanced ccRCC	• mPFS: 7.4 mo (5.5–10.9) • ORR: 35%	HCRN GU16-260, NCT03117309 [164]
Nivo ± followed by Ipi	II	First- or second-line and salvage, advanced ccRCC and non-ccRCC	Nivo with salvage Ipi (69% of patients in arm B): • Conversion from SD/PD to PR: 4% (90% CI, 1–11)	OMNIVORE, NCT03203473 [166]
Nivo ± followed by Nivo + Ipi	II	First- or second-line and salvage, metastatic or unresectable non-ccRCC	• mPFS: 4.0 mo (95% CI, 3.6–7.4) • ORR: 17%	UNISON/ANZUP 1602, NCT03177239 [167]
Nivo followed by Nivo + Ipi “boost”	II	First- and second-line, intermediate/high-risk advanced RCC	Nivo first-line vs. second-line: • mOS: 27.2 mo (95 % CI, 19.9–NE) vs. 20.2 mo (95 % CI, 15.6–NE) • ORR: 28 % vs. 17 %	TITAN-RCC, NCT02917772 [165]
Durva + Treme	II	ICI/CD137/cMet-naïve and VEGF treatment-refractory (advanced ccRCC) or VEGF treatment naïve or -refractory (advanced pRCC)	• mPFS: 4.9 months (95% CI, 2.5–10.0)	CALYPSO, NCT02819596
Durva + Treme	Ib	Neoadjuvant and adjuvant, locally advanced RCC		NCT02762006 [163]
MEDI0680 (PD-1) + Durva vs. Nivo	I/II	ICI-naïve, advanced ccRCC	MEDI0680 + Durva vs. Nivo: • mPFS 3.6 mo vs. 3.6 mo • ORR: 14.3% vs. 19.0%	NCT02118337 [171]
Nivo + Ipi, Relatlimab (LAG-3), BMS-986205 (IDO1), or BMS-813160 (CCR2/CCR5)	II	First- or second-line, advanced RCC	Nivo + Ipi: • ORR: 15.2%	FRACTION-RCC, NCT02996110 [168]
Relatlimab ± Nivo	I/II	ICI-naïve, RCC		NCT01968109
LAG525 (LAG-3) ± Spartalizumab (PD-1)	I/II	Second- or later-line, advanced RCC		NCT02460224 [169]
Sabatolimab (TIM-3) ± Spartalizumab	I-Ib/II	ICI-naïve or pre-treated, advanced RCC		NCT02608268 [170]
INCAGN02390 (TIM-3)	I	Later-line, advanced RCC		NCT03652077
CA-170 (PD-L1, PD-L2, VISTA)	I	ICI-ineligible, advanced RCC		NCT02812875 [172]
TKIs + ICIs				
Bev + Atezo	III	First-line, PD-L1+ metastatic RCC	Bev + Atezo vs. Sunitinib: • mPFS (PD-L1+): 11.2 mo vs. 7.7 mo [HR 0.74; 95% CI, 0.57–0.96; *p* = 0.0217] • mOS (ITT): [HR 0.93; 95% CI, 0.76–1.14]	IMmotion151, NCT02420821 [29]
Cabo + Atezo	III	Second- or third-line, ICI-refractory advanced RCC		CONTACT-03, NCT04338269 [180]
Nivo + Ipi ± Cabo	III	First-line, intermediate/poor-risk advanced ccRCC		COSMIC-313, NCT03937219 [178]
Nivo + Ipi followed by maintenance Nivo (CR), Cabo (PD), or Nivo ± Cabo (non-CR/PD)	III	First-line, intermediate/poor-risk advanced ccRCC		A031704/ PDIGREE, NCT03793166 [179]
Nivo ± Ipi ± followed by Nivo or Sunitinib/Pazopanib	II	First-line, metastatic ccRCC stratified into one of four molecular subtypes		BIONIKK, NCT02960906 [143]
Savolitinib + Durva	II	ICI/CD137/cMet naïve and VEGF treatment refractory (advanced ccRCC) or VEGF treatment naïve or refractory (advanced pRCC)	• mPFS (pRCC): 4.9 mo (95% CI, 2.5–10.0) • mPFS (pRCC, MET-driven disease): 10.5 mo (95% CI, 2.9–15.7) • mOS (pRCC, MET-driven disease: 27.4 mo (95% CI, 7.3–NR) • ORR (pRCC): 29% • ORR (pRCC, MET-driven disease): 57%	CALYPSO, NCT02819596 [181]
Sitravatinib + Nivo	II	Neoadjuvant, ccRCC	• ORR 11.8% (33.3% for 120 mg Sitravatinib)	NCT03680521 [174]
Cabo + Atezo	I/II	First-line (ccRCC) or prior VEGF TKI treatment (non-ccRCC)	40 mg Cabo + Atezo vs. 60 mg Cabo + Atezo: • mPFS (ccRCC): 19.5 mo (95% CI, 11.0 –NE) vs. 15.1 mo (95% CI, 8.2–22.3) • ORR (ccRCC): 53% (80% CI, 41–65) vs. 58% (80% CI, 46–70) 40 mg Cabo: • ORR (non-ccRCC): 31% (80% CI, 20–44) • mPFS (non-ccRCC): 9.5 mo	COSMIC-021, NCT03170960 [175]
Cabo + Pembro	I/II	First- or later-line Pembro/Cabo-naïve, advanced RCC	• mPFS: 10.4 mo (95% CI, 6.3–NR) • mOS: NR • 17.8 mo ORR: 60% (95% CI, 0.458–1.00)	NCT03149822 [177]
Lenvatinib + Pembro	Ib/II	First- or later-line, metastatic ccRCC	ICI-pre-treated population: • mOS: NR (95% CI, NR–NR) • mPFS: 12.2 mo (95% CI, 9.5–17.7) • 24 wk ORR: 55.8% (95% CI, 45.7–65.5)	KEYNOTE-146, NCT02501096 [182]
Ibrutinib + Nivo	Ib/II	Second- or later-line, advanced RCC		NCT02899078 [173]
Cabo + Nivo ± Ipi	I	Later-line, advanced ccRCC		NCT02496208 [176]
Cellular Therapies				
VEGFR2 CAR T cells	I/II	Second- or later-line, metastatic RCC		NCT01218867
Anti-c-Met CAR T cells	I/II	PR/NR/recurrency if prior ICI, c-Met+ RCC		NCT03638206
ROR2, AXL CAR T cells	I/II	ROR2+ or AXL+ relapsed and refractory stage IV metastatic RCC		NCT03393936
CD70 CAR T cells	I/II	Second- or later-line, CD70+ ccRCC		NCT02830724 †
D-CIK + Axitinib	II	First-line or after progression on anti-angiogenesis or cytokine therapy, advanced RCC		NCT03736330
HIF-2α + ICI				
PT2385 + Nivo or Cabo	I	Second- or later-line, advanced ccRCC	PT2385 + Nivo: • mPFS: 7.3 mo • ORR: 22%	NCT02293980 [184]
Vaccines				
IMA901 + Sunitinib	III	First-line, advanced ccRCC	IMA901 + Sunitinib vs. Sunitinib: • mOS: 33.17 mo (95% CI, 27.8–41.4) vs. NR (33.7–NR) [HR 1.34; 95% CI, 0.96–1.86; *p* = 0.087]	IMPRINT, NCT01265901 [187]
Rocapuldencel-T + Sunitinib	III	First-line, advanced RCC	Rocapuldencel-T + Sunitinib vs. SOC: • mOS: 27.7 mo (95% CI, 23.0–35.9) vs. 32.4 mo (95% CI, 22.5-NE) [HR 1.10; 95% CI, 0.83–1.40] • mPFS: 6.0 mo vs. 7.83 mo [HR 1.15; 95% CI, 0.92–1.44] • ORR: 42.7% (95% CI, 37.1–48.4) vs. 39.4% (95% CI, 31.6–47.5)	ADAPT, NCT01582672 † [189]
AGS-003 + Sunitinib	II	First-line, intermediate/poor-risk metastatic ccRCC	• mOS: 30.2 mo (95% CI, 9.4–57.1) • mPFS: 11.2 mo (95% CI, 6.0–19.4)	NCT00678119 [188]
INTUVAX/Ilixadencel (intra-tumoral) + Sunitinib	II	Neoadjuvant + adjuvant first-line, synchronous metastatic RCC	INTUVAX + Sunitinib vs. Sunitinib: • mPFS 11.8 mo vs. 11.0 mo • ORR 42.4% vs. 24.0% • mDOR 7.1 mo vs. 2.9 mo	MERECA, NCT02432846 [191]
GEN-009 Adjuvanted Vaccine + Nivo or Pembro	I/II	First-line (intermediate/poor-risk beginning Nivo + Ipi) or after anti-angiogenic therapy (beginning nivolumab), advanced RCC		NCT03633110 [192]
VB10.NEO + Bempegaldesleukin	I/IIa	PR/SD/PD on ICI, advanced ccRCC		DIRECT-01, NCT03548467 [193]
Pexastimogene Devacirepvec + Cemiplimab	I/II	First- or later-line ICI-naïve, advanced ccRCC	• ORR: 37.5%	NCT03294083 [194]
DC Tumor Fusion + GM-CSF	I/II	Chemotherapy- and biological therapy-naïve, stage IV RCC		NCT00458536
Autologous or Allogeneic tumor cells	I/II	Chemotherapy-refractory, metastatic RCC		NCT00722228
Treme + Durva + PolyICLC	I/II	Dual ICI-naïve, biopsy-accessible advanced RCC		NCT02643303 [195]
COMBIG-DC/INTUVAX	I	Intermediate/poor-risk metastatic RCC	• mOS: NR	NCT01525017 [190]
Neovax ± Ipi	I	First- or later-line ICI-naïve, stage III/IV resectable ccRCC		NCT02950766
Cevumeran ± Atezo	Ia/Ib	First- or later-line ICI-naïve, advanced RCC		NCT03289962
PSMA plasmid DNA vaccine	I	Favorable-risk RCC		NCT00096629
IL-2 + ICI				
Bempegaldesleukin + Nivo	III	First-line, advanced ccRCC		PIVOT-09, NCT03729245 [199]
High Dose IL-2 + Pembro	II	First- or later-line ICI-naïve, metastatic RCC	• Projected ORR: 69%	NCT02964078 [196]
High Dose IL-2 + Nivo	Ib/II	First-, second-, or third-line, IL-2-, IFN-, PD-1/PD-L1-ICI-naïve, metastatic ccRCC		NCT02989714 [197]
Bempegaldesleukin + Nivo ± Ipi	I/II	First-, second-, or third-line IL-2-naïve, advanced RCC		PIVOT-02, NCT02983045 [198]
Microbiome				
± Deferred cytoreductive nephrectomy following Nivo + Ipi (microbiome analysis)	III	First-line, intermediate/poor-risk synchronous metastatic RCC		NORDIC-SUN, NCT03977571
Nivo or Pembro ± Metformin or Rosiglitazone (microbiome analysis)	II	PD-1/L1 ICI-naïve, advanced RCC		NCT04114136
Nivo + Ipi ± SBRT (microbiome analysis)	II	First-line, intermediate/poor-risk metastatic RCC		CYTOSHRINK, NCT04090710
FMT from ICI-responding donors + ICI	I/II	Receiving or eligible for ICI, advanced RCC		TACITO, NCT04758507
FMT + Nivo + Ipi (irAEs analysis)	I	First-line, intermediate/poor-risk advanced RCC		PERFORM, NCT04163289
MRx0518 (*Enterococcus*)	I	No therapy in past 2 years, RCC		MICROBIOME, NCT03934827
ICI/s ± other systemic therapy (microbiome analysis)	N/A	Eligible for ICI, stage I–IV RCC		PARADIGM, NCT05037825
Nivo ± Ipi, Durva ± Treme (microbiome analysis)	N/A	ICI-naïve, advanced RCC		NCT04107168
Other Immunomodulators				
Epacadostat (IDO1) + Pembro	III	First-line, advanced ccRCC		KEYNOTE-679/ECHO-302, NCT03260894
Entinostat (HDAC) + Nivo + Ipi	II	Nivo + Ipi-refractory, metastatic RCC		NCT03552380
High Dose IL-2 ± Entinostat	II	Second- or third-line (including ICI), advanced ccRCC		NCT03501381
Atezo + Bev + Entinostat	I/II	ICI-naïve (II Cohort A) or ICI-refractory (II Cohort B), metastatic RCC	• mPFS: 7.6 mo (95% CI, 1.6–16.3) • ORR: 47.1% (95% CI, 23.0–72.2)	NCT03024437 [203]
Aldesleukin + Entinostat	I/II	First-, second-, or third-line, metastatic ccRCC	• ORR: 37% (90% CI, 24–51) [*p* = 0.010] • mPFS 13.8 mo (95% CI, 6.0–18.8) • mOS 65.3 mo (95% CI, 52.6–65.3) • Decreased Tregs associated with response	NCT01038778 [204]
HBI-8000 (HDAC) + Nivo	I/II	Advanced RCC		NCT02718066
NIR178 (A2AR) + Spartalizumab	II	Later-line, TKI-refractory, advanced RCC		NCT03207867
Dalantercept (ALK1/TGF-β) + Axitinib	II	Second- or third-line (including VEGF inhibitor) ICI-naïve, advanced ccRCC	Dalantercept + Axitinib vs. Axitinib + Placebo: • mOS: NR vs. NR [HR 1.39; 95% CI, 0.70–2.77; *p* = 0.349] • mPFS: 6.8 mo vs. 5.6 mo [HR 1.11; 95% CI, 0.71–1.73; *p* = 0.670] • ORR: 19.0% (95% CI, 9.9–31.4) vs. 24.6% (95% CI, 14.5–37.3)	DART, NCT01727336 [200,201]
Oleclumab (CD73) + Durva	II	No prior CD73/CD39/innate immune agonist, advanced RCC		DOMINATION, NCT04262375
Axitinib ± Ivuxolimab (OX40)	II	Second- (one prior TKI + ICI) or third-line (one prior non-axitinib TKI, one ICI), metastatic RCC		NCT03092856
INBRX-106 (OX40) ± Pembro	I	Later-line OX40 agonist-naïve, advanced RCC		NCT04198766
Feladilimab (ICOS) + Treme	I/II	CTLA-4/ICOS-treatment-naïve, advanced ccRCC		NCT03693612 [209]
Varlilumab (CD27) + Nivo	I/II	Anti-angiogenic therapy-experienced, ICI-naïve, no CTLA-4/CD27 therapy in past 3 mo, advanced ccRCC		NCT02335918
INCAGN01876 (GITR) + Nivo or Ipi or Nivo + Ipi	I/II	Later-line, advanced RCC		NCT03126110
Axitinib ± Carotuximab (Endoglin)	I/II	Later-line, one prior TKI (other than axitinib) ± one prior ICI, advanced ccRCC		NCT01806064 [202]
Telaglenastat (glutaminase) + Nivo	I/II	Later-line ± ICI-naïve, advanced ccRCC		NCT02771626
Utomilumab (4-1BB) + Pembro	I	Advanced RCC		KEYNOTE-0036, NCT02179918 [208]
Mogamulizumab (CCR4)	I	RCC		NCT02946671
Dostarlimab (PD-1) + Niraparib (PARP) Cobolimab (TIM-3), Bev, or Platinum-based doublet chemotherapy	I	Advanced RCC		IOLite, NCT03307785 [205]
Valemetostat (EZH1/2) + Ipi	I	Later-line ICI- and anti-angiogenic therapy-refractory, advanced ccRCC		NCT04388852
Ciforadenant (A2AR)	I	Second- or third-line ICI-refractory, ccRCC		NCT02655822 [206,207]
Siremadlin (MDM2) + Spartalizumab	I	Later-line, advanced RCC		NCT02890069
Nivo + Stereotactic Body Radiotherapy	II	Second- or third-line PD-1/L1/L2-naïve, advanced RCC	• mOS: 22.1 mo (95% CI, 18.1-NR) • mPFS: 4 mo (95% CI, 2.8–7.1) • ORR: 19%	NIVES, NCT03469713 [211]
Guadecitabine + Durva	I/II	ICI-naïve (Cohort 1) or PD-1/L2-regractory (Cohort 2), advanced ccRCC	• mOS: NR • mPF: 17 mo	NCT03308396 [210]
Treme ± Cryoablation	N/A	CTLA-4 ICI-naïve, advanced ccRCC or non-ccRCC		NCT02626130

Immune checkpoint inhibitor (ICI), tyrosine kinase inhibitor (TKI), nivolumab (Nivo), ipilimumab (Ipi), pembrolizumab (Pembro), atezolizumab (Atezo), durvalumab (Durva), tremelimumab (Treme), bevacizumab (Bev), cabozantinib (Cabo), stereotactic body radiation therapy (SBRT), standard of care (SOC), no evidence of disease (NED), months (mo), weeks (wk), disease free survival (DFS), median overall survival (mOS), confidence interval (CI), not estimable (NE), hazard ratio (HR), median progression free survival (mPFS), overall response rate (ORR), median duration of response (mDOR), complete response (CR), partial response (PR), stable disease (SD), progressive disease (PD), not reached (NR), intention-to-treat (ITT), immune-related adverse events (irAE), not applicable (N/A), and milligrams (mg). * Data from published literature or abstracts. † Trials discontinued or suspended.

## 5. Conclusions

To optimize the therapeutic landscape and future clinical trial design for RCC patients, it is essential to understand the basic and translational tumor immunology science that laid the foundation for these treatment breakthroughs [212]. CD8 and CD4 TILs infiltrate the majority of RCC tumors, and while cytolytic activity may be high, they often display an exhausted phenotype with expression of immune checkpoints extending past PD-1 and CTLA-4. TAMs are also abundant within the TME, often with an immunosuppressive skewing, activating Tregs and causing T cell dysfunction. Tumor cells themselves promote immune escape, upregulating WNT/β-catenin signaling, inducing MDSC differentiation and NK cell dysfunction, and exhibiting metabolic reprogramming. Many ongoing trials target these players, combining PD-1/PD-L1 or CTLA-4 ICIs with various other checkpoint-based antagonists, immunostimulatory agonists, chemokines/cytokines, and genetic and metabolic modulators, among other agents. Data on TKIs have revealed numerous immunomodulatory roles, with the specific effects between different TKIs not yet well characterized. Also underappreciated is the impact of the gut microbiome on distal anti-tumor immunity, and further delineation of the responsible mechanisms may provide more specific targets for RCC immunomodulation. In addition, there are multiple emerging techniques that may aid in further characterizing the immune response and relevant biomarkers in RCC, including analysis of circulating tumor cells, single-cell sequencing, and ex vivo organoid modeling [213].

Analyses of post-ICI treatment specimens have provided insight into the immunomodulatory effects of these therapies, and the potential mechanisms of both response and resistance. Extreme ICI responders have been noted to have strong CD8 T cell infiltration both in pre- and post-treatment tissue [214], and data from trials have demonstrated ICIs linked to increased TILs, expression of IFNγ-stimulated and Th1 genes, MHC-I levels, and chemokines such as CXCL9, CXCL10, and CX3CL1 [97,215]. Responding patients had differentially increased expression of lymphoid and myeloid gene sets, IFNγ response genes, and checkpoints including TIGIT, CTLA-4, PD-L2, as well as decreased TGF-β signaling and MMP3 expression, with RIG-I-MDA5 pathway activity noted in non-responders with a high degree of TILs [129]. Single-cell transcriptomic analysis identified increased activated and terminally exhausted CD8 T cells and pro-inflammatory TAM skewing in ICI responders, as well as two distinct tumor cell populations with differential responses to ICI [94]. Clinical trials that incorporate post-treatment biopsies will continue to aid in our understanding of the mechanisms of response and resistance to immunotherapy.

Many uncertainties still remain within RCC, including the surprisingly low mutational burden for an immunotherapy-responsive cancer. The high indel rate resulting in the generation of more immunogenic neo-epitopes partially addresses this phenomenon, yet the number of observed neo-antigens remains lower than expected, and the paradoxical correlations between TMB, TILs, and immunotherapy response are still not well understood. Potential genetic and molecular links, such as the lack of *PBRM1* mutations, enrichment for 9p21.3 loss, and increased RIG-I-MDA5 pathway activity may explain ICI non-response in CD8 T cell-infiltrated tumors. Moving forward, these concepts may prove crucial for understanding the interactions in tumor immune microenvironment, optimizing response to current therapies, and innovating the next generation of immunomodulatory agents personalized to individual patient characteristics.
